# Which out-of-office measurement technique should be used for diagnosing hypertension in prehypertensives?

**DOI:** 10.1038/s41371-019-0284-x

**Published:** 2019-11-07

**Authors:** Şükrü Ulusoy, Gülsüm Özkan, Mustafa Arıcı, Ülver Derici, T. Akpolat, Şule Şengül, Rahmi Yılmaz, Şehsuvar Ertürk, Yunus Erdem, on behalf of the Turkish Society of Hypertension and Renal Diseases

**Affiliations:** 1 Department of Nephrology, School of Medicine, Karadeniz Technical University, Trabzon, Turkey; 2 Department of Nephrology, School of Medicine, Namık Kemal University, Tekirdağ, Turkey; 3 Department of Nephrology, School of Medicine, Hacettepe University, Ankara, Turkey; 4 Department of Nephrology, School of Medicine, Gazi University, Ankara, Turkey; 5 Department of Nephrology, Liv Hospital, Istinye University, Istanbul, Turkey; 6 Department of Nephrology, School of Medicine, Ankara University, Ankara, Turkey

**Keywords:** Hypertension, Kidney diseases

## Abstract

Hypertension (HT) is diagnosed with high office blood pressure (BP), although confirmation with the addition of out-of-office measurements is currently recommended. However, insufficient data are available concerning the use of out-of-office BP measurement techniques for the diagnosis of HT in the prehypertensive population. The aim of the present study was to determine which out-of-office measurements yielded earlier and more frequent detection of development of HT in prehypertensive patients. Two hundred seven prehypertensive patients under monitoring in the Cappadocia cohort were included in the study. Office BP was measured five times at 1-min intervals, followed by 24-h ambulatory BP monitoring (24-h ABPM). Home BP measurement (HBPM) was performed five times, at the same times in the morning and evening, at 1-min intervals for 1 week. The same procedure was carried out at 4–6-month intervals for ~2 years. HT was diagnosed in 25.6% of subjects, masked HT in 11.1%, and white coat HT in 2.9%, while 23.7% remained prehypertensive and 36.7% became normotensive. Briefly, 56.6% of the patients with HT were diagnosed with office plus 24-h ABPM, 13.2% with office plus HBPM, and 30.2% with office plus HBPM and 24-h ABPM. Office with 24-h ABPM yielded statistically significantly more diagnoses (*p* < 0.001). In conclusion, our prospective observational study evaluated the usefulness of out-of-office BP measurements in confirming diagnosis of HT in prehypertensive patients. The findings show that 24-h ABPM detected HT earlier and more frequently in this high-risk population.

## Introduction

Hypertension (HT) is a widespread global health problem, the prevalence of which is increasing, and one with high morbidity and mortality. The general prevalence of HT in the 2018 European Society of Cardiology (ESC) and European Society of Hypertension (ESH) guideline is ~30–45% [1].

The diagnosis and treatment of HT depends on accurate blood pressure (BP) measurement. Reliable BP measurement is performed with intra-arterial BP measurement. However, since this method is effectively impossible in clinical practice, BP is measured using noninvasive methods. Office BP is the most commonly employed BP measurement technique [2]. The disadvantages of diagnosing HT solely in the office setting include measurement errors, the limited number of measurements that can be conveniently taken, and the confounding risk for isolated clinical HT [3, 4]. Almost all HT guidelines therefore state that diagnosis of HT should be confirmed with repeated office BP measurements, or out-of-office BP measurements (home BP monitoring (HBPM) or 24-h ambulatory BP monitoring (24-h ABPM)) [1, 5, 6]. Numerous studies have shown that 24-h ABPM is the gold standard in confirming diagnosis of HT, and that HBPM yields more reliable results than office BP measurement. The two out-of-office BP measurement methods have also been proved to be more reliable than office BP measurement in predicting cardiovascular events (CVEs) [7, 8].

Studies have compared HT diagnosis confirmation rates using consecutive office BP measurements, 24-h ABPM, or HBPM, in patients with high office BP. These have reported that office and HBPM are not as reliable as 24-h ABPM in diagnosing HT [9–11]. However, since 24-h ABPM is not available in all clinics and due to concerns over costs, the confirmation of diagnosis of HT in the event of high office BP using one of the out-of-office BP measurement methods appears now to be generally accepted [1, 5, 6]. In addition, there is no explicit information and advice concerning the use of out-of-office BP measurement in the screening and follow-up of HT in patients with optimal and prehypertensive BP values. The recently published 2018 ESH/ESC guideline recommends confirmation with out-of-office BP measurement due to potential suspicion of masked HT in individuals with elevated BP (SBP 130–139 mmHg, DBP 85–89 mmHg) [1].

BP values between optimal BP and stage 1 HT have historically been classified using such terminology as “transient HT,” “borderline HT,” “high-normal BP,” and “prehypertension.” Irrespective of the range and terminology, the development of HT and cardiovascular morbidity and mortality are accepted as being more common in this patient group compared with the optimal BP group [12–14].

The aim of our prospective cohort study was to show which of 24-h ABPM and HBPM measurements in addition to office BP measurement revealed development of HT earlier and more frequently in patients diagnosed as prehypertensive with office BP and followed-up for ~2 years.

## Material and method

### Cappadocia cohort

This prospective cohort study is being conducted by the Turkish Society of Internal Medicine (TIHUD) [15]. The observational component commenced in March 2013. Following receipt of informed written consent, subjects’ baseline data were collected using a 167-question electronic questionnaire. This produced information concerning demographic characteristics, lifestyle, and medical history, including diagnosed illnesses and medication use. All subjects underwent detailed physical examinations at which BP (at least twice), body weight, height, and waist circumferences were measured. BP measurement at time of enrollment in the cohort was performed in line with Joint National Committee 7 (JNC 7) guideline [16]. All participants are monitored annually in terms of changes in these parameters, onset of new illnesses, changes in weight or waist circumference, medication use, smoking status, alcohol consumption, and dietary characteristics. Nonpharmacological measures had been proposed to prehypertensive patients due to ethical necessity.

### Patient selection

**Fig. 1:**
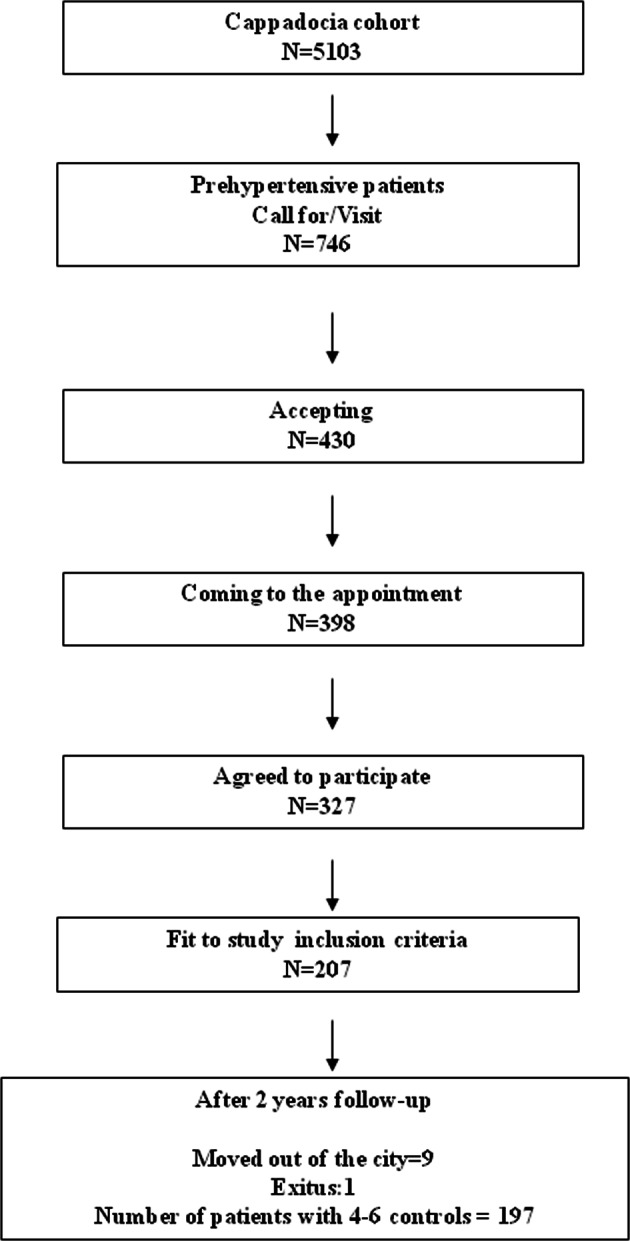
Patient selection flow chart for the study

Two years after the start of the cohort follow-up (January 2015), prehypertensive individuals identified based on two office measurements in 2013 from among 5150 individuals under monitoring in the Cappadocia cohort were randomly invited to participate in the study. Three hundred twenty-seven individuals agreed to participate, and 207 meeting the inclusion criteria at the first office visit and fully completing the stages of the study protocol at the first visit were enrolled (Fig. 1). Individuals aged over 18, who had been informed about the study and expressed verbal willingness to take part, and with sufficient intellectual capacity to provide a medical history, to measure BP at home and to perform 24-h ABPM, were enrolled. Pregnant women, and patients with known heart failure, kidney failure, or chronic liver disease, using antihypertensive drugs, or refusing to provide contact details were excluded. Ethical committee approval for the study was obtained from the Hacettepe University Medical Faculty, Turkey. The study commenced following receipt of informed written consent from subjects.

### Study protocol

Selection of the 207 individuals agreeing to take part in the study, based on BP measurements performed during inclusion in the cohort 2 years previously, was carried out in line with the JNC 7 guideline. Patients with an office BP measurement ≥ 140/90 mmHg, and a 24-h ABPM all-day average ≥ 130/80 mmHg, and/or a home BP measurement (HBPM) ≥ 135/85 mmHg were diagnosed with HT. Patients with office BP values of 120–139/80–89 mmHg were regarded as prehypertensive. Patients who were normotensive at office BP measurement but in whom HT was identified at HBPM and/or 24-h ABPM were regarded as having masked HT, while those determined as hypertensive at office measurement but normotensive at HBPM and at 24-h ABPM were evaluated as having white coat HT (WCHT) [16].

Following recording of demographic data at the first office visit, the 207 subjects included in the study underwent detailed physical examinations. Height, weight, and waist circumference were measured. Office BP was measured in accordance with the JNC 7 guideline [16]. One-week HBPM was then performed, followed by 24-h ABPM. Office, home, and 24-h ABPM measurements were repeated once every 4–6 months during the follow-up process. Patients diagnosed with HT at any visit were monitored in line with the JNC 7 guideline [16]. Hypertensive patients were referred to family medicine practitioners for evaluation, treatment, and follow-up.

### Office BP measurement

Office BP was measured using a UA-651SL monitor (A&D Company, 1-243 Asahi, Kitamoto-shi, Saitama-ken.364-8585 Japan), a validated device. Before the procedure, all patients were asked to rest for at least 5 min in a relaxed position in a quiet room at a comfortable temperature. We asked the patients whether they had consumed any caffeine, alcohol, or cigarettes in the previous 30–60 min. BP was measured by a physician from both arms using a cuff of a suitable size for the patient’s upper arm, with the upper arm held at heart level, with the back and the upper arm supported, and with the patient sitting upright. We were careful to ensure that patients did not cross their legs or speak during the procedure. Once BP had been measured from both arms, subsequent BP measurements were carried out using the arm eliciting the highest value. BP was measured five times at 1-min intervals. The first measurement was excluded from the analysis. The mean value of the next four measurements was recorded as office BP. Office BP measurement was repeated following the same procedure at each visit every 4–6 months.

### Home BP measurement

An UA-651SL monitor (A&D Company, 1-243 Asahi, Kitamoto-shi, Saitama-ken.364-8585 Japan) device was used for HBPM. Patients measured their own home BP after receiving the appropriate training. BP was measured five times at 1-min intervals every morning and evening for 1 week, in line with current guideline recommendations [1]. At the end of that period, mean morning and evening values were calculated and recorded as home BP values. HBPM was repeated following the same procedure at each visit every 4–6 months, and the devices used were randomized.

### Ambulatory BP measurement

A Mobil-O-Graph NG 24 h ABPM Classic (I.E.M. GmbH, Stolberg, Germany) device was used to measure 24-h ABPM. Patients recorded times spent sleeping, waking, and eating, together with daily activities performed. Sleeping–waking periods were evaluated accordingly. Patients were also asked to ensure that the arm was kept immobile during BP measurement. Daytime BP measurement was performed at 15-min intervals and night-time measurement at 30-min intervals. Subjects with at least 70% measurement records for 24-h ABPM were included in the analysis [1]. Measurements were performed using the same procedure at each visit every 4–6 months, and the ABPM devices were randomized.

### End point

Office BP measurement ≥ 140/90 mmHg at any visit from the first, while HBPM was ≥135/85 mmHg or mean daytime 24-h ABPM was ≥130/80 mmHg, was defined as HT. Patients with HBPM < 135/85 mmHg and mean daytime 24-h ABPM < 130/80 mmHg despite office BP measurements ≥ 140/90 mmHg throughout the study were regarded as WCHT, and patients with office BP measurement < 140/90 mmHg and HBPM ≥ 135/85 mmHg and/or mean daytime 24-h ABPM ≥ 130/80 mmHg were regarded as masked HT.

### Statistical analysis

PASW 18.0 for Windows software was employed for statistical analysis. Descriptive statistics were expressed as number and percentage for categorical variables and as mean, standard deviation, and median (minimum–maximum) for numerical variables. Since normal distribution conditions were not established for the numerical variables of age and BMI, the Mann–Whitney U test was used in the comparison of groups with HT, prehypertension, and normotension over 24 months, while the chi-square test was used to compare the categorical variable of gender. *p* values lower than 0.05 were regarded as statistically significant.

## Results

The Cappadocia cohort study involving 5150 individuals commenced in 2013, and 327 subjects with prehypertension identified with office BP measurement at the start of the cohort agreed to participate in the present study. Two hundred seven prehypertensive subjects capable of measuring BP with office BP, HBPM, and 24-h ABPM were finally included.

The median age of the 207 subjects in the study was 50 years, and 62.3% were women. Two years previously in the initial cohort, the average office BP of the same 207 individuals was 127.76 ± 6.53/76.04 ± 7.59 mmHg. Baseline demographic parameters of the entire study population and demographic data of normotensive, prehypertensive, and hypertensive patients at the end of the study are shown in Table 1. A median number of five [4–6] visits were performed over a mean follow-up period of 23.30 ± 1.75 months, with an average time between visits of 4.31 ± 1.26 months. After the first control, nonpharmacological measures were proposed to prehypertensive patients for ethical reasons.

**Table 1:** Baseline demographic parameters of whole study population and demographic data of normotensive, prehypertensive, and hypertensive patients at the end of the study

	Baseline	At the end of the study		
	Whole population (*n* = 207) Mean ± SD	Normotensive (*n* = 76) Mean ± SD	Prehypertensive (*n* = 49) Mean ± SD	Hypertensive (*n* = 53) Mean ± SD
BMI (kg/m^2^)	29.94 ± 4.96	28.39 ± 4.8	30.88 ± 5.03	31 ± 4.51
Office SBP (mmHg)	122.60 ± 14.24	109.97 ± 4.55	125.01 ± 6.37	142.5 ± 10.33
Office DBP (mmHg)	78.25 ± 9.14	70.25 ± 4.78	79.29 ± 6.13	90.98 ± 7.82
Home SBP (mmHg)	116.33 ± 11.69	109.29 ± 5.88	117.05 ± 8.85	130.22 ± 10.84
Home DBP (mmHg)	72.63 ± 7.32	69.18 ± 4.07	71.96 ± 6.05	79.11 ± 7.05
24-h-ABPM all-day SBP (mmHg)	120.16 ± 11.90	106.61 ± 9.13	112.07 ± 15.48	122.9 ± 22.83
24-h-ABPM all-day DBP (mmHg)	75.48 ± 8.36	67.24 ± 5.53	73.66 ± 6.96	89.82 ± 12.53

*BMI* body mass index, *24-h ABPM* 24-h ambulatory blood pressure monitoring, *SBP* systolic blood pressure, *DBP* diastolic blood pressure

At the end of 2 years, HT was determined in 25.6% of subjects, while 23.7% remained prehypertensive and 36.7% became normotensive. The BP measurement methods by which patients were diagnosed with HT and distribution by HT classifications at the end of the study are presented in Table 2. As shown in Table 2, WCHT was observed at a rate of 2.9% and masked HT at 11.1%.

**Table 2:** Distribution of HT classification of patients and the method by which they are determined

	Method	*N*: 207 (%)
HT	Office with 24-h-ABPM (*n* = 30)	53 (25.60%)
	Office with HBPM (*n* = 7)	
	Office with 24-h ABPM and HBPM (*n* = 16)	
Masked HT	24-h ABPM (*n* = 19)	23 (11.1%)
	HBPM (*n* = 1)	
	HBPM with 24-h ABPM (*n* = 3)	
White coat HT		6 (2.9%)
Prehypertension		49 (23.7%)
Normotension		76 (36.7%)

*HT* hypertension, *24-h ABPM* 24-h ambulatory blood pressure monitoring, *HBPM* home blood pressure monitoring

**Fig. 2:**
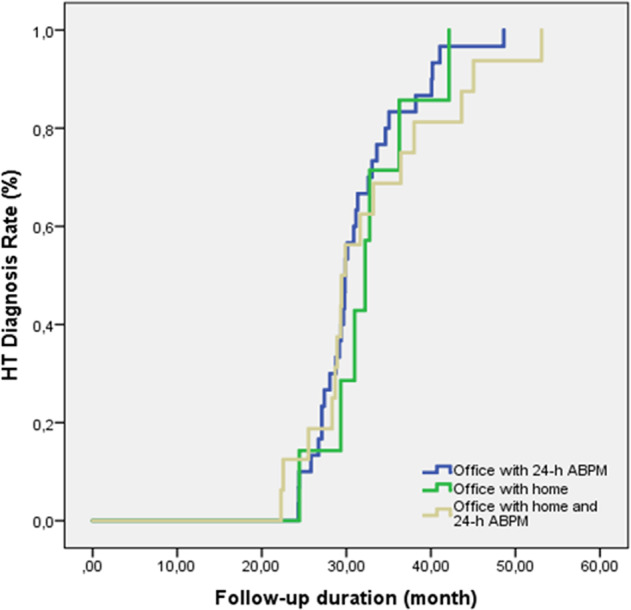
HT detection times and rates of the methods used

Thirty (56.6 %) of the 53 patients diagnosed with HT were diagnosed with office and 24-h ABPM, seven (13.2%) with office and HBPM, and 16 (30.2%) with office plus HBPM and 24-h ABPM (Table 2). Statistically significantly more diagnoses were made with office plus 24-h ABPM (*p* < 0.001). HT was detected in 29 patients at the first control, 12 patients at the second, six patients at the third, four patients at the fourth, and two patients at the sixth. At initial controls, 62.1% of the 29 patients were diagnosed HT with office BP plus 24-h ABPM, and 58.3% of the 12 patients were diagnosed at the second control. Diagnosis rates were significantly higher with this method than with office plus HBPM or office plus HBPM and 24-h ABPM (*p* = 0.001) (Fig. 2).

## Discussion

In this prospective, observational, cohort study, we examined and followed-up 207 subjects diagnosed as prehypertensive. We used automated devices for measuring BP under both home and office conditions and also for 24-h ABPM. At the end of the follow-up, HT was diagnosed in 25.6% of subjects, masked HT in 11.1%, and WCHT in 2.9%, while 23.7% remained prehypertensive and 36.7% became normotensive. HT was diagnosed more frequently and earlier with office plus 24-h ABPM than office plus HBPM or office plus HBPM and 24-h ABPM. Some of the prehypertensive individuals may have become normotensive after complying with recommendations regarding lifestyle changes made for ethical reasons at each control.

The prevalence of HT, irrespective of age, is ~32%, while the Turkish Society of Hypertension and Renal Diseases cites a figure of 30.3% in Turkey [1, 5, 6, 16, 17]. A mean 4-year HT incidence rate of 21.4% was determined in the HinT study [18]. Although ours was not an incidence study, we determined HT in 25.6% of subjects in the 4-year period from the beginning of the cohort study and our 2-year prospective follow-up. The greater incidence of HT in our research than in the 4-year HinT study may be due to our study involving a prehypertensive patient group. Several previous studies have shown that prehypertensives frequently develop HT. Selassie at al. monitored 18,865 nonhypertensive individuals for 7 years and determined development of HT in 63.8%. Presence of prehypertension emerged as a significant predictor of development of HT [19]. In the TRial Of Preventing HYpertension study, HT developed in 40.4% of prehypertensive patients monitored with lifestyle modification in the second year and in 63% at the end of 4 years [14]. Studies have also shown that in addition to HT development, CVEs are also more common in prehypertensive individuals [12, 13].

The difficulty with HT is not limited to its high prevalence. Another major problem is that various controversial issues regarding the diagnosis of HT have still not been resolved. One particular problem involves BP measurement being correctly performed for diagnosis of HT. Unfortunately, levels of BP measurement and of correct measurement are both quite low worldwide [3, 4]. Office BP measurement is the oldest method of diagnosing HT, but for reasons such as low levels of correct BP measurement, and the inability to determine conditions such as WCHT and masked HT using office BP, the use of out-of-office methods is recommended in order to confirm diagnosis [1, 2, 5]. While the majority of HT guidelines recommend the use of out-of-office BP measurement methods, particularly under some specific conditions, the NICE guideline was the first to emphasize the role of 24-h ABPM in confirming diagnosis of HT [6]. In later years, other HT guidelines also began recommending the use of out-of-office BP measurement methods in confirming diagnosis of HT [1, 5]. Previous studies and the majority of guidelines recommend the use of out-of-office BP measurements in individuals with high BP. However, there are no explicit recommendations concerning the use of out-of-office BP measurements in the screening and follow-up of HT in the prehypertensive population, with its high risk of HT development. However, due to the high incidence of masked HT in individuals with high-normal BP, the 2018 ESH/ESC guideline recommended out-of-office BP measurement in these individuals [1]. The purpose of our study was to investigate the place of out-of-office BP measurements in the early diagnosis of HT in the monitoring of a prehypertensive population. Insufficient data are available on this subject in the literature. Our study showed that more patients were diagnosed with the out-of-office BP measurement methods 24-h ABPM and HBPM in a prehypertensive population with high rates of HT development and CVEs. The low number of patients diagnosed with HT made it difficult to compare the times of diagnosis between different methods. However, considering the number of patients diagnosed with HT with office BP plus 24-h ABPM, especially in the first two controls, we concluded that HT can be diagnosed earlier with office plus 24-h ABPM. In addition, 24-h ABPM was more effective than HBPM in detecting masked HT and WCHT.

The reported prevalence of WCHT in several previous studies is ~13–35% [20–23]. Studies investigating cardiovascular-related and all-cause mortality and WCHT have determined a minimal increase in mortality in patients with WCHT [24, 25]. The incidence of WCHT in our study was 2.9%. This figure being lower than those in other studies may be due to the higher number of office BP measurements in our study [20–23]. The majority of studies determining the prevalence of WCHT have been performed using the average of two consecutive office BP measurements, while in our study five measurements were performed at 1-min intervals at every visit, with the first measurement being discarded and the average of the remaining values being recorded.

The reported prevalence of masked HT in population studies is 10–26% [21, 26, 27]. The rate in our study was 11.1%, which is compatible with the previous literature. In contrast to WCHT, there are studies showing that masked HT causes an increased cardiovascular risk as high as that in sustained HT [24, 26, 28]. We determined a not inconsiderable rate of masked HT with 24-h ABPM. We conclude that 24-h ABPM is more effective in this population with a high risk of developing HT and of CVEs.

There are a number of limitations to our prospective observational cohort study. One is that only office BP measurement was performed at the beginning of the cohort. However, one strength of our study is that we continued to perform all three measurements 4–6 times over the last 2 years. The patient number of 207 in the cohort initiated among a wide population derives from our selection of a prehypertensive population and in particular to the low number of patients willing to have 24-h ABPM performed 4–6 times. The low number of patients diagnosed with HT prevented us from performing various analyses and reduced the significance of some findings that might otherwise have been more significant.

In conclusion, this prospective observational cohort study demonstrates the role of out-of-office BP measurements in confirming diagnosis of HT in the prehypertensive group, a subject on which sufficient data are lacking. Our findings show that more HT was detected with 24-h ABPM in the screening and follow-up of HT in this high-risk population. In addition, we observed that ABPM is more useful than home measurement in the diagnosis of masked HT, another important problem. We think that other studies performed in the light of our research will further strengthen the place of 24-h ABPM in the diagnosis of HT in the prehypertensive group.

### Summary table

#### What is known about topic

- The diagnosis and treatment of HT depends on accurate BP measurement.
- Office BP is the most commonly employed BP measurement technique.
- The disadvantages of diagnosing HT solely in the office setting include measurement errors, the limited number of measurements that can be conveniently taken, and the confounding risk for isolated clinic HT.
- Almost all HT guidelines therefore state that diagnosis of HT with repeated office BP measurements, or out-of-office BP measurements (HBPM or 24-h ABPM) should be confirmed.
- In addition, there is no explicit information and advice concerning the use of out-of-office BP measurement in the screening and follow-up of HT in patients with optimal and prehypertensive BP values.

#### What this study adds

- Our prospective observational study evaluated the usefulness of out-of-office BP measurements in confirming diagnosis of HT in prehypertensive patients.
- We showed that 24-h ABPM detected HT earlier and more frequently in this high-risk population.
